# Regional innovation distribution and its dynamic evolution: Policy impact and spillover effect—Based on the perspective of innovation motivation

**DOI:** 10.1371/journal.pone.0235828

**Published:** 2020-07-10

**Authors:** Yanyang Yan, Zhichao Wu

**Affiliations:** College of Finance and Statistics, Hunan University, Changsha, China; Shandong University of Science and Technology, CHINA

## Abstract

This study aims to explore the direct effect and spillover effect of innovation policy in the distribution and dynamic evolution of the regional innovation from the perspective of innovation motivation using the spatial econometric model. Substantive innovation and strategic innovation in one region could affect innovation in another region. In addition, the direct effect and spatial spillover effect of innovation policy could significantly affect innovation; however, they exert a higher impact on substantive innovation. Considering different policy tools, we found that government subsidies exert a significant positive impact on substantive innovation and strategic innovation, whereas financial institution loans exert a significant negative impact on substantive innovation and strategic innovation. And for the impact range, the government subsidies are higher than that of financial institution loans. Furthermore, this study reveals the leading environmental factors affecting regional innovation and provide a policy basis to promote the construction of an innovation-oriented country.

## 1. Introduction

The 19^th^ National Congress of the Communist Party of China report officially declared that China’s socialist construction has entered a new era. In the economic construction, “China’s economy has transitioned from the stage of high-speed growth to the stage of high-quality development, marking as the critical period of transforming the development mode, optimizing the economic structure, and transforming the growth power.” Regarding how to change the development mode, the report highlighted that innovation should be used to drive economic development. However, innovation warrants massive continuous capital investment [1], and the ambiguity of innovation income makes its capital source and quantity limited. Conversely, there exists a “free-riding” phenomenon in the technology innovation to damage the interests of innovators. To alleviate this dilemma, the government supports innovation activities through relevant incentive policies, especially subsidies, financial support and tax preference which could directly decrease the cost and risk of innovation, enhance the return rate of innovation, and then stimulate innovation vitality. Second, the typical activities of one region, especially its advanced experience, could soon be served as an example for others to follow and become a leading role [2,3]. Owing to China’s vast territory, the internal and external conditions of all regions differ; thus, the innovation policy could also differ, leading to the phenomenon of policy transmission and spillover, which affects the innovation activities in adjacent regions.

For innovation behavior, previous studies classify innovation behavior from innovation content or innovation intensity, and few studies classify innovation behavior from the perspective of innovation motivation. However, besides innovation aimed at promoting technological advancement and maintaining competitive advantage, some innovation activities also aim at other interests, which is a strategic behavior [4]. Strategic innovation is just a strategy of enterprise management; its purpose is not to substantially enhance the technical competitiveness of the enterprise but obtain some benefits [5]. Strategic innovation often caters to the government policies and regulations [6,7] and values quantity rather than the quality of innovation. Therefore, different motivation of innovation may have different responses to the direct effect and spillover effect of innovation policy and it is of great significance to study different motivation of innovation for better evaluation of the implementation effect of policies. Unlike previous studies, this study investigates the direct effect and spillover effect of innovation policy from the perspective of innovation motivation. The main research questions explored in this study are as follows: (i) Whether the direct effect and spillover effect of innovation policy will change in different types of innovation? (ii) What are the differences in the effects of different types of innovation policies?

The remainder of this paper is organized as follows: Section 2 provides literature review; Section 3 provides the data applied to construct the models and the construction of spatial econometric models; Section 4 presents the distribution of innovation and innovation policy; Section 5 presents the empirical results and robust analysis; and Section 6 provides the main conclusions and enlightenment of this study.

## 2. Literature review

Since the technological development and knowledge accumulation are crucial factors that determine economic growth, innovation has been extensively researched. Hu and Mathews [8,9] proposed that the national innovation capacity is the fundamental driving force for economic operation. Whether it is the optimization and upgrading of economic structure or the transformation of development mode, the fundamental development power could be attributed to the innovation, and innovation ability is the strongest weapon for sovereign countries to augment and win the economic globalization competitiveness [10,11]. The production efficiency and competitiveness of a country can be enhanced by constantly improving the country’s innovation ability. With the increasingly frequent exchanges between regions, the relations among regions, countries, and even the whole world are improved [12–16]. Reportedly, the spatial dependence of innovation makes the spatial knowledge spillover the main factor to affect the innovation distribution [17–19]. When knowledge creation ability, relevant technical resources, and human resources are deficient, it becomes even more crucial to enhance the ability of technology innovation through technology diffusion. In other words, the comprehensive effect of knowledge creation and knowledge spillover is the real driving force to improve the regional technology innovation [20]. Among them, technological distance, knowledge gap, academic ability, economic development level, and opening degree are considered the essential factors that affect the regional knowledge spillover. Moreover, the geographical proximity causes the innovation cluster, hence, it promotes the enhancement of regional innovation level [21–28]. As a crucial factor affecting regional innovation, the role of policy cannot be overlooked. Blind [29] assessed the comprehensive impact of competition, price, product, environment, patent protection, legislation, and other policies on innovation, using the data of OECD (Organization for Economic Cooperation and Development) countries, and established the significant role of most of the policies. Fabrizio et al. [30] discussed that demand policies affect technology transfer between the origin country and the destination country. Rioja and Valev [31] examined the correlation between financial development and technological innovation in 74 countries and deduced that policies play a positive role in promoting innovation in high financial development regions, whereas no link was identified between policies and innovation in low financial development regions. As the innovation output–related input exhibits different spillover effects [12,32], policies can not only directly affect the supported regions but also they could affect nearby regions through technology spillover or resource redistribution.

To date, limited studies have focused on the impact of policy from the perspective of innovation motivation, especially put the direct effect and spatial spillover effect of innovation policy into a unified analysis framework from the perspective of innovation motivation. Typically, innovators default to input resources in R&D activities, resulting in technological progress and competitive advantage, which is manifested as high-quality innovation behaviour [5]. However, sometimes, the innovation activities of innovators could be presented as a strategic innovation behaviour [4,33,34]. Thus, innovators could choose different innovation behaviors depending on their own conditions and environment. To gain market share, technological progress, product upgrading [35], and competitive advantage, innovators tend to choose high-quality innovation behaviour, which is substantive innovation. The purpose is not to gain technological development and product upgrading but obtain more subsidies through simple innovation [36] or pursuing the quantity of innovation [34]. Besides, enterprises intend to gain more reputation through the number and speed of innovation, they are more willing to execute simple innovation to avoid the loss of the company’s interests due to the failure of R&D or the uncertainty of innovation revenue. And this is strategic innovation. Regarding policy, on the one hand, the support of innovation policy to innovation is to decrease the uncertainty of innovation. On the other hand, the support of innovation policy to innovation is to make up for the loss caused by innovation being imitated. Thus, this study aims to explore the following: (i) What are the differences between the direct effects of innovation policies on substantive innovation and strategic innovation? (ii) What are the differences between spillover effects of innovation policies on substantive innovation and strategic innovation?

Accordingly, this study aims to investigate the differences between the direct effect and spillover effects of innovation policy in two types of innovation from the perspective of innovation motivation. In addition, as different types of policy could affect the impact of policy implementation, this study differentiates different policy types.

## 3. Data and modeling

### 3.1 Variables

#### 3.1.1 Measuring innovation

In previous studies, there are many indicators to measure innovation, such as patents, research and development(R&D) expenditure, new product sales, and the number of new product development projects to measure innovation. Feldman and Florida [37] reported a high correlation between innovation and patents, reaching 0.934. In this study, we used patents to measure innovation—a popular approach also used in some recent studies [30,32,38–41]. In addition, patents represent the successful resource integration and team cooperation, which are successful innovation, and R&D expenditure could be uneconomical [42,43]. Second, R&D could be debating if it should be capitalized or expensed according to accounting norms [44]. Moreover, the new product sales and the number of new product development projects have not been disclosed until 2009, and nearly 50% of region-year observations have missing values. And only the industrial enterprises above designated size would be censused these two indicators. Therefore, new product sales, and the number of new product development projects cannot fully represent the level of regional innovation. Hence, patents are better indicators to measure innovation. In China, patents are classified into three types—invention, utility model, and design patent. Based on the quality of innovation, the invention patent is recognized as substantive innovation, while utility model and design patent are recognized as strategic innovation [4,5].

#### 3.1.2 Measuring innovation policy

As innovation has a high probability of failure, its investment might not be recovered. Conversely, even if innovation is successful, innovators might not be able to completely monopolize its benefits because of the spillover effect of innovative technologies and products. Thus, the government supports innovation activities through relevant incentive policies, enhancing the contribution of the public sector to innovation performance [45]. Generally, innovation policy can be divided into supply-oriented policy, demand-oriented policy and environment-oriented policy according to different policy tools [46], and this method has been widely used [47,48]. In China, supply-oriented policy and environment-oriented policy are preferred, in which government subsidies in supply-oriented policy and financial support and tax preference in environment-oriented policy are more used [49]. As a technology catching-up country, China’s government usually provides government subsidies as a key tool to encourage enterprises to innovate independently [36]. Government subsidies can not only directly insert economic resources into micro-enterprises and transfer the innovation risks of high-tech companies but also efficiently promote the production factors and economic resources flowing to the R&D activities of micro-enterprises. Moreover, government subsidies could significantly affect R&D investment, profit, and sales with new products [50–53]. Besides, innovation activities usually encounter serious external financing constraints owing to the uncertainty of return, information asymmetry, moral hazard, and other problems in the innovation investment [54], this is a major barrier for enterprises that take the initiative in innovation, especially small and medium-sized enterprises [55,56]. Furthermore, financial institutions loans could offer financial support for enterprises, ease financing constraints, and promote the prosperity of science and technology and financial development. As for, the tax preference, on the one hand, it can reduce the risk of innovation failure by reducing the tax rate, and the same time, it can internalize the economic benefits of innovation activities to promote the company to increase innovation investment [57]. However, the tax preference for regional innovation has not been disclosed. Accordingly, we chose government subsidies and financial institutions loans as innovation policies and analysed the policy effects of different types of innovation policies.

#### 3.1.3 Control variables

Besides the innovation policy mentioned above, some variables could also affect regional innovation. In this study, we considered other indicators, including scientific and technological personnel input, regional economic development level, worker quality, and degree of opening to the outside world, as control variables [29,32,58–60], which are represented by the R&D personnel of full-time equivalent, per-capita GDP, years of education, imports, and exports, respectively.

The per capita GDP, imports and exports are all adjusted at the constant prices in 2001. Meanwhile, both exports and imports were converted into renminbi (RMB) based on the average exchange rate between RMB and US dollar. We adjusted government subsidies and financial institution loans through a composite index weighted using the 2001 Consumer Price Index and the Fixed Asset Investment Index, according to the purposes of innovative expenditures. Zhu and Xu [61] reported that the weights of the Consumer Price Index and the Fixed Asset Investment Index in the weighted composite index were 0.55 and 0.45, respectively. Moreover, the years of education were obtained by multiplying the proportion of each type of population by the corresponding years of education. The indicators mentioned above were derived from the China statistical yearbook of science and technology and China statistical yearbook. Of note, the research duration of this paper was 2001–2018.

### 3.2 Spatial econometric model

Based on Anselin’s research “almost all spatial data have the characteristics of spatial dependence or spatial autocorrelation” [62], and ignoring such spatial correlation could lead to the bias of model setting. Using the spatial econometric model, we reflected the real situation more objectively.

In the spatial econometric model, the spatial weight matrix plays a vital role, which suggests the connection between one region and other regions. Usually, the spatial weight matrix can be determined by the adjacency or spatial distance of the spatial unit. However, the adjacency fails to precisely depict the spatial correlation between regions (Not only the adjacent spatial units will influence each other, but also the non-adjacent spatial units will influence each other). Thus, the spatial weight matrix was based on the spatial distance in this study. Typically, the spatial weight matrix based on the distance definition is presented as follows:

$$w_{ij}=\{\begin{array}{c} \frac{1}{d_{ij}},i\neq j\\ 0,i=j \end{array}$$ (1)

$$w_{ij}=\{\begin{array}{c} 1,d_{ij}\leq d\\ 0,d_{ij}>d \end{array}$$ (2)

In Eq (1), when *i* ≠ *j*, the spatial weight between regions *i* and *j* is the reciprocal of their distance. When *i* = *j*, the space weight between regions *i* and *j* is 0. In the Eq (2), when the distance between regions *i* and *j* is less than or equal to a given distance *d*, the spatial weight is 1; when the distance between regions *i* and *j* is greater than the given distance *d* the spatial weight is 0. To avoid the deviation caused by the subjective selection of a given distance *d*, we constructed the spatial weight according to Eq (1).

Usually, spatial econometric models are primarily divided into the spatial autoregressive model (SAR) and spatial error model (SEM), based on different representation positions of the spatial correlation. In the SAR model, the explained variables of the assumed region *i* not only depend on their own independent variables but also could depend on the explained variables of their neighbours. The model was as follows:

$$\text{ln}\;pat_{it}=\rho \sum_{j=1}^{n}w_{ij}\text{ln}\;pat_{it}+\beta_{1}\text{ln}\;gov+\beta_{2}\text{ln}\;fin_{it}+\sum_{k}\delta_{k}x_{kit}+\mu_{it}$$ (3)

where *pat*_*it*_ is innovation (when different innovation motives are distinguished, it represents substantive innovation and strategic innovation), *ρ* is the spatial autocorrelation coefficient, $\sum_{j=1}^{n}w_{ij}$ represents the row standardized spatial weight matrix, *gov*_*it*_, *fin*_*it*_ are innovation policies, *x*_*kit*_ is a series of control variables, including the regional economic development level (lngdp), expressed by the logarithm of regional per capita GDP. The regional opening degree (lnexp, lnimp) is expressed by logarithm of regional exports and imports. Regional scientific and technological personnel input (lnpeo) is expressed by the logarithm of regional R&D equivalent full-time personnel. Regional worker quality (eduyear) is expressed by years of education in the region, and *δ* is the coefficient of the corresponding control variables. *μ*_*it*_ is the random error term.

In the SEM model, when some sudden changes occur in a region, the impact is transmitted to the neighbouring region in some form, and the transmission has a long-time continuity and attenuation. The model was as follows:

$$\text{ln}\;pat_{it}=\beta_{1}\text{ln}\;gov_{it}+\beta_{2}\text{ln}\;fin_{it}+\sum_{k}\delta_{k}x_{kit}+\mu_{it}$$ (4)

$$\mu_{it}=\lambda \sum_{j=1}^{n}w_{ij}\mu_{it}+\varepsilon_{it}$$ (5)

where *λ* is the spatial error coefficient, *ε*_*it*_ are random interference item. The meaning of the remaining variables is the same as above.

### 3.3 Spatial spillover effect

Based on the complex dependence between variables in the spatial econometric model, the model coefficient cannot be simply considered as real elasticity. Thus, decomposition and estimation of the direct and indirect effects (i.e., spatial spillover effect) need to be used by the model suggested elsewhere [63,64]. The direct effect measures the effect of the independent variable change on the dependent variable in the region, while the indirect effect (spatial spillover effect) measures the effect of the change of one-unit independent variable on other spatial unit dependent variables. The specific calculation is as follows.

Firstly, the general form of SAR model is defined as follows:

Y=ρWY+Xβ+μ (6)

Suppose *μ* ~ *N*(0, *σ*^2^*I*_*n*_), convert the general form of spatial autoregressive model into the following form:

$$(I_{n}-\rho W)Y=X\beta +\mu$$ (7)

then

$$Y={\left(I_{n}-\rho W\right)}^{-1}X\beta +{\left(I_{n}-\rho W\right)}^{-1}\mu$$ (8)

among them (*I*_*n*_ − *ρW*)^−1^ = *I*_*n*_ + *ρW* + *ρ*^2^*W*^2^ + *ρ*^3^*W*^3^ + ⋯.

Suppose *X* contains *P* explainable variables, and the *r*th explainable variable was *x*_*r*_ = (*x*_1*r*_, *x*_2*r*_, …, *x*_*Nr*_), then $X\beta =\left(x_{1},x_{2},\ldots ,x_{p}\right){\left(\beta_{1},\beta_{2},\ldots ,\beta_{p}\right)}^{\prime }=\overset{p}{\underset{r=1}{\sum }}\beta_{r}x_{r}$.

Thus, the Eq (8) can be written as

$$Y=\sum_{r=1}^{P}\beta_{r}{\left(I_{n}-\rho W\right)}^{-1}x_{r}+{\left(I_{n}-\rho W\right)}^{-1}\mu =\sum_{r=1}^{P}S_{r}(W)x_{r}+{\left(I_{n}-\rho W\right)}^{-1}\mu$$ (9)

among them *S*_*r*_(*W*) = *β*_*r*_(*I*_*n*_ − *ρW*)^−1^

Thus, Eq (9) can be transformed into a matrix:

$$\left(\begin{array}{c} Y_{1}\\ Y_{2}\\ \vdots \\ Y_{N} \end{array}\right)=\sum_{r=1}^{P}\left(\begin{array}{cccc} S_{r}{\left(W\right)}_{11} & S_{r}{\left(W\right)}_{12} & \cdots & S_{r}{\left(W\right)}_{1N}\\ S_{r}{\left(W\right)}_{21} & S_{r}{\left(W\right)}_{22} & \cdots & S_{r}{\left(W\right)}_{2N}\\ \vdots & \vdots & \cdots & \vdots \\ S_{r}{\left(W\right)}_{N1} & S_{r}{\left(W\right)}_{N2} & \cdots & S_{r}{\left(W\right)}_{NN} \end{array}\right)\left(\begin{array}{c} x_{1r}\\ x_{2r}\\ \vdots \\ x_{Nr} \end{array}\right)+{\left(I_{n}-\rho W\right)}^{-1}\mu$$ (10)

among them, *S*_*r*_(*W*)_*ij*_ is the (*i*, *j*) element of *S*_*r*_(*W*).

According to Eq (10), we can see that:

$$\frac{\partial Y_{i}}{\partial X_{ir}}=S_{r}{\left(W\right)}_{ii}$$ (11)

$$\frac{\partial Y_{i}}{\partial X_{jr}}=S_{r}{\left(W\right)}_{ij}$$ (12)

Eqs (11) and (12) represent the direct effect of the variable *X*_*ir*_ of region *i* on the interpreted variable *Y* of region *i* and the spatial spillover effect of the variable *X*_*jr*_ of region *j* on the interpreted variable *Y* of region *i*, respectively.

## 4. Innovation and innovation policy distribution

Before the empirical analysis, we first make a preliminary analysis of the distribution of innovation and innovation policies to understand their distribution characteristics.

### 4.1 Innovation distribution

We created statistics on the accumulation innovation in different regions during 2001–2018 to identify the distribution of innovation in different regions of China.

As shown in Table 1, in terms of innovation during 2001–2018, the top three provinces included Jiangsu, Guangdong, and Zhejiang, accounting for 17.55%, 15.53%, and 11.78% of the innovation, respectively. In addition, innovation of the top five provinces reached 57.28% of the national level. The last five provinces included Inner Mongolia, Ningxia, Hainan, Qinghai, and Tibet, accounting for only 0.72% of the innovation. The findings suggested that China’s innovation output is primarily concentrated in the eastern region, while regions with low innovation output are mostly concentrated in the western region of China.

**Table 1:** Distribution of innovation from 2001 to 2018.

Province	Innovation	Percentage (%)	Province	Innovation	Percentage (%)
Jiangsu	4583502	17.55	Hebei	418039	1.60
Guangdong	4057021	15.53	Jiangxi	352430	1.35
Zhejiang	3076143	11.78	Heilongjiang	318643	1.22
Shandong	1774713	6.80	Guangxi	311550	1.19
Beijing	1467556	5.62	Guizhou	205910	0.79
Shanghai	1210779	4.64	Shanxi	181955	0.70
Anhui	1097682	4.20	Yunnan	178574	0.68
Sichuan	1029597	3.94	Jilin	167066	0.64
Fujian	805198	3.08	Gansu	140359	0.54
Henan	757911	2.90	Xinjiang	105987	0.41
Hubei	754885	2.89	Inner Mongolia	87167	0.33
Tianjin	704532	2.70	Ningxia	45687	0.17
Shaanxi	589612	2.26	Hainan	32244	0.12
Liaoning	551182	2.11	Qinghai	20773	0.08
Hunan	550960	2.11	Tibet	5590	0.02
Chongqing	533401	2.04			

Unit: piece. Innovation is measured by patent (invention, utility model and design patent). Calculated according to the data of each year in China Science and Technology Statistical Yearbook.

In addition, China is further divided into the following seven regions: North China (Beijing, Tianjin, Hebei, Shanxi, and Inner Mongolia), Northeast China (Liaoning, Jilin, and Heilongjiang), East China (Shanghai, Jiangsu, Zhejiang, Anhui, Fujian, Jiangxi, and Shandong), central China (Henan, Hubei, and Hunan), South China (Guangdong, Guangxi, and Hainan), Southwest China (Chongqing, Sichuan, Guizhou, Yunnan, and Tibet), and Northwest China (Shaanxi, Gansu, Qinghai, Ningxia, and Xinjiang). When the provinces are divided into the regions listed above, the proportions of each region’s innovation in the national innovation is to measure the innovation degree of each region. The number of innovations in East China, South China, and North China accounted for 49.40%, 16.85%, and 10.95% of the national level, respectively. Moreover, the contribution of these three regions reached up to 77.20%, of which the contribution of East China reached 49.40%, which is far more than that of other regions. Furthermore, the contribution of Northwest China was only 3.46%, of which Shaanxi Province contributed 2.26%, while the contribution of other provinces was less than 1%. Accordingly, innovation displays apparent polarization phenomenon in China.

**Fig 1:**
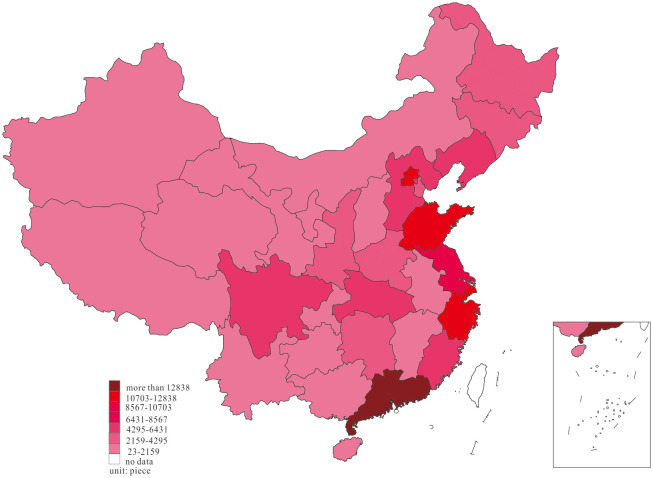
Distribution of innovation, in 2001.

**Fig 2:**
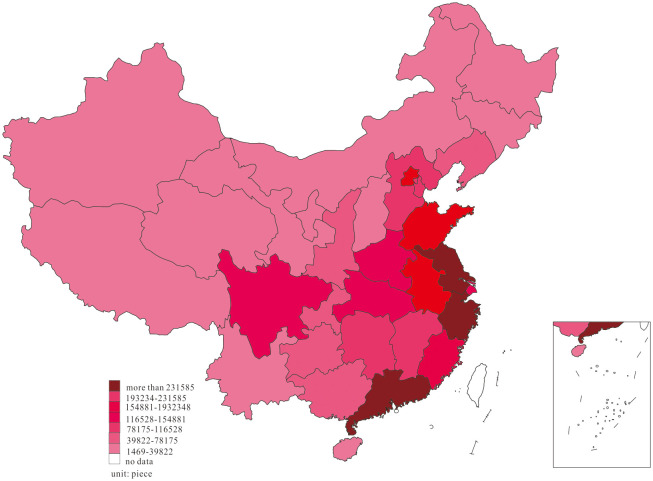
Distribution of innovation, in 2018.

We further investigated the innovation distribution at two-time endpoints of the research interval to examine the dynamic change of innovation, and the results are shown in Figs 1 and 2.

Based on the dynamic process of innovation changes shown in Figs 1 and 2, we found that most of the innovations in 2001 were primarily distributed in the east region of Guangdong, Zhejiang, Shanghai, Beijing, Shandong, Jiangsu, and Fujian, in the middle region of Hubei, Hunan, and Henan, as well as Sichuan and Shaanxi in the west. In 2018, China’s innovation witnessed a phenomenon of agglomeration, in which the innovation in Anhui, Jiangxi, Chongqing, Guizhou, and Guangxi increased considerably, forming agglomeration with adjacent high-output areas.

Based on the innovation motivation, the distribution of substantive innovation and strategic innovation was further analysed as follows.

As shown in Table 2, regarding substantive innovation between 2001 and 2018, the top three provinces included Jiangsu, Guangdong, and Beijing, accounting for 16.23%, 13.13%, and 9.77% of substantive innovation, respectively. In addition, substantive innovation of the top five provinces reached 54.00% of the national level. The last five provinces, including Inner Mongolia, Ningxia, Hainan, Qinghai, and Tibet, accounted for only 0.74% of substantive innovation. The findings suggested that China’s substantive innovation was mainly concentrated in the eastern region, while the regions with low substantive innovation were mainly concentrated in the western region of China.

**Table 2:** Distribution of substantive innovation from 2001 to 2018.

Province	Substantive innovation	Percentage (%)	Province	Substantive innovation	Percentage (%)
Jiangsu	1356822	16.23	Fujian	165252	1.98
Guangdong	1098011	13.13	Heilongjiang	106923	1.28
Beijing	817351	9.77	Hebei	101686	1.22
Zhejiang	635786	7.60	Guizhou	72497	0.87
Shandong	607984	7.27	Shanxi	64995	0.78
Anhui	500998	5.99	Jilin	64058	0.77
Shanghai	489891	5.86	Jiangxi	63581	0.76
Sichuan	327297	3.91	Yunnan	56773	0.68
Hubei	274146	3.28	Gansu	44016	0.53
Tianjin	233528	2.79	Xinjiang	25053	0.30
Shaanxi	225489	2.70	Inner Mongolia	22948	0.27
Henan	214536	2.57	Ningxia	17352	0.21
Liaoning	214147	2.56	Hainan	12266	0.15
Hunan	188138	2.25	Qinghai	7394	0.09
Guangxi	185865	2.22	Tibet	1674	0.02
Chongqing	165737	1.98			

Unit: piece. Substantive innovation is measured by invention patent. Calculated according to the data of each year in China Science and technology statistical yearbook.

Likewise, when China was divided into seven regions, the number of substantive innovations in East China, South China, and North China accounted for 45.69%, 15.50%, and 14.83% of the national total, respectively. In addition, the contribution of these three regions reached up to 76.02%, of which the contribution of East China reached 45.69%, which is far higher than that of other regions. The contribution of Northwest China was only 3.82%, of which Shaanxi Province contributed 2.70%, while the contribution of other provinces was less than 1%. Thus, substantive innovation revealed noticeable polarization phenomenon in the country.

We applied a similar analysis in the distribution of strategic innovation.

As shown in Table 3, in terms of strategic innovation during 2001–2018, the top three provinces included Jiangsu, Guangdong, and Zhejiang, accounting for 18.17%, 16.67%, and 13.75% of strategic innovation, respectively, this was more centralized distribution compared with substantive innovation. In addition, strategic innovation of the top five provinces reached 59.22% of the national level. The last five provinces, including Inner Mongolia, Ningxia, Hainan, Qinghai, and Tibet, accounted for only 0.73% of strategic innovation. The findings suggested that China’s strategic innovation was primarily concentrated in the eastern region, while the regions with low substantive innovation were mainly in the western region of China. Moreover, the number of strategic innovation in East China, South China, and North China accounted for 51.14%, 17.49%, and 9.12% of the national total, respectively; furthermore, the contribution of these three regions reached up to 77.75%, of which the contribution of East China reached 51.14%, which is far greater than that of other regions. The contribution of Northwest China was only 3.28%, of which Shaanxi Province contributed 2.70%, while the contribution of other provinces was less than 1%. Thus, strategic innovation exhibited apparent polarization phenomenon in the country.

**Table 3:** Distribution of strategic innovation from 2001 to 2018.

Province	Strategic innovation	Percentage (%)	Province	Strategic innovation	Percentage (%)
Jiangsu	3226680	18.17	Hebei	316353	1.78
Guangdong	2959010	16.67	Jiangxi	288849	1.63
Zhejiang	2440357	13.75	Heilongjiang	211720	1.19
Shandong	1166729	6.57	Guizhou	133413	0.75
Shanghai	720888	4.06	Guangxi	125685	0.71
Sichuan	702300	3.96	Yunnan	121801	0.69
Beijing	650205	3.66	Shanxi	116960	0.66
Fujian	639946	3.60	Jilin	103008	0.58
Anhui	596684	3.36	Gansu	96343	0.54
Henan	543375	3.06	Xinjiang	80934	0.46
Hubei	480739	2.71	Inner Mongolia	64219	0.36
Tianjin	471004	2.65	Ningxia	28335	0.16
Chongqing	367664	2.07	Hainan	19978	0.11
Shaanxi	364123	2.05	Qinghai	13379	0.08
Hunan	362822	2.04	Tibet	3916	0.02
Liaoning	337035	1.90			

Unit: piece. Strategic innovation is measured by utility model and design patent. Calculated according to the data of each year in China Science and Technology Statistical Yearbook.

**Fig 3:**
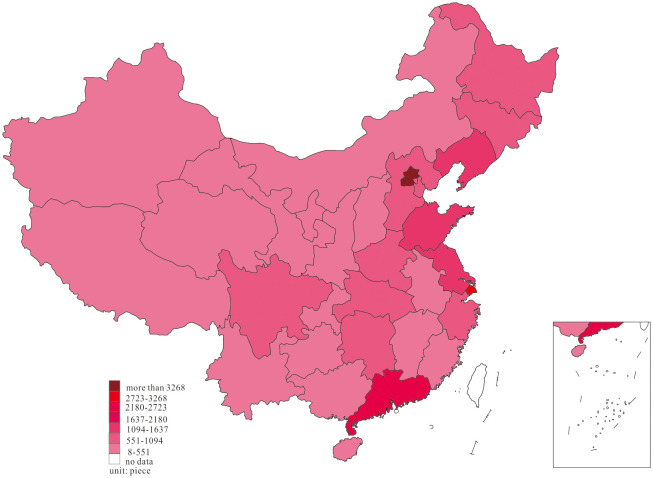
Distribution of substantive innovation, in 2001.

**Fig 4:**
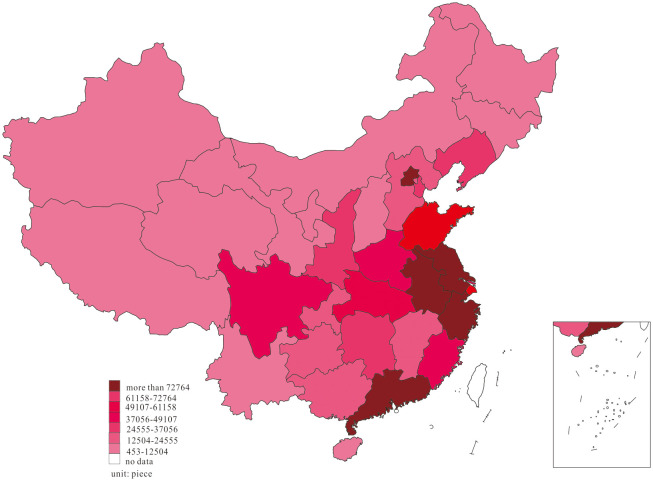
Distribution of substantive innovation, in 2018.

**Fig 5:**
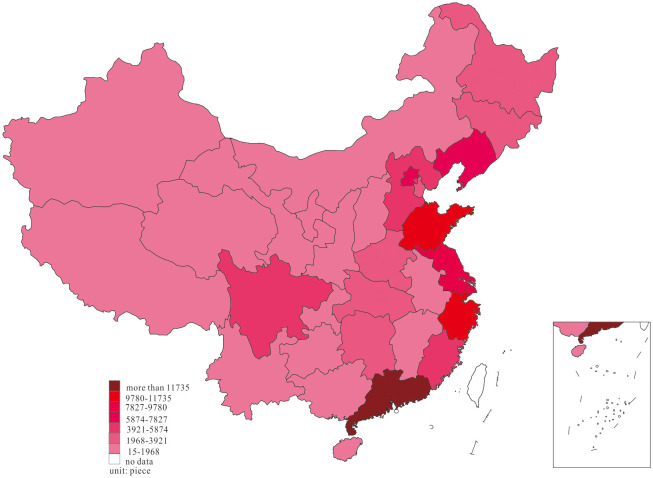
Distribution of strategic innovation, in 2001.

**Fig 6:**
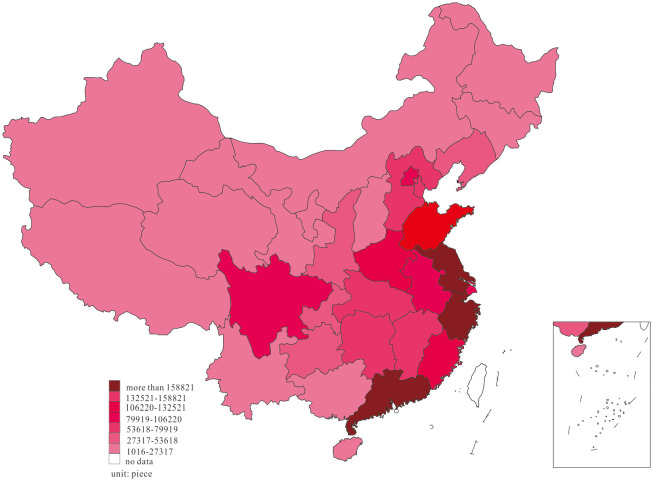
Distribution of strategic innovation, in 2018.

We further investigated substantive innovation and strategic innovation distribution at the two-time endpoints of the research interval to note the dynamic change of substantive innovation and strategic innovation, and the results are shown in Figs 3–6.

Based on the dynamic process of substantive innovation shown in Figs 3 and 4, we observed that most of the substantive innovation in 2001 was mainly distributed in the East region of Beijing, Shanghai, Guangdong, Liaoning, Jiangsu, Shandong, and Zhejiang, in the middle region of Hubei, Hunan, and Henan, as well as Sichuan in the west. In 2018, the phenomenon of agglomeration was observed in China’s substantive innovation, in which substantive innovation in Anhui, Shaanxi, Chongqing, Guangxi, and Guizhou increased markedly, forming agglomeration with adjacent high-output areas. Figs 5 and 6 show that most of strategic innovation in 2001 was primarily distributed in the East region of Guangdong, Zhejiang, Shanghai, Jiangsu, Beijing, and Fujian, in the middle region of Hunan, Henan, and Hubei, as well as Sichuan in the west. In 2018, the phenomenon of agglomeration was noted in China’s strategic innovation, in which strategic innovation in Anhui, Jiangxi, Chongqing, Shaanxi, and Guizhou increased significantly, forming agglomeration with adjacent high-output areas. Furthermore, the development of substantive innovation and strategic innovation revealed the formation of innovation clusters.

### 4.2 Innovation policy distribution

We performed a similar analysis of the innovation policies during 2001–2018 and obtained statistics based on the two indicators of government subsidies and financial institution loans.

Table 4 shows that, in terms of government subsidies intensity during 2001–2018, the top three provinces included Beijing, Shanghai, and Shaanxi, accounting for 27.66%, 10.11%, and 7.00% of the total subsidies, respectively. In addition, the top five provinces accounted for 57.54%, among which Beijing alone accounted for the highest proportion, which was significantly higher than other provinces and cities. The bottom five provinces were Xinjiang, Hainan, Ningxia, Qinghai, and Tibet, with government subsidies at 1.16% of the national level. The findings suggested that government subsidies were mainly concentrated in the eastern region of China, which is similar to substantive innovation and strategic innovation.

**Table 4:** Distribution of government subsidies from 2001 to 2018.

Province	Government subsidies	Percentage (%)	Province	Government subsidies	Percentage (%)
Beijing	76759878	27.66	Jilin	4498618	1.62
Shanghai	28048043	10.11	Fujian	3545001	1.28
Shaanxi	19418628	7.00	Chongqing	3209035	1.16
Sichuan	19345339	6.97	Yunnan	3093224	1.11
Jiangsu	16104712	5.80	Gansu	2818293	1.02
Guangdong	14198205	5.12	Jiangxi	2542696	0.92
Hubei	10190608	3.67	Shanxi	2529682	0.91
Liaoning	10167048	3.66	Guangxi	2497596	0.90
Shandong	9327968	3.36	Inner Mongolia	1602252	0.58
Zhejiang	8086282	2.91	Guizhou	1442948	0.52
Tianjin	7406614	2.67	Xinjiang	1297313	0.47
Anhui	7161532	2.58	Hainan	701102.2	0.25
Heilongjiang	5868545	2.11	Ningxia	557547.9	0.20
Hebei	5034266	1.81	Qinghai	450754.6	0.16
Henan	4737232	1.71	Tibet	217880.9	0.08
Hunan	4640093	1.67			

Unit: ten thousand yuan. Calculated according to the data of each year in China Science and Technology Statistical Yearbook.

Table 5 shows that, in terms of financial institution loans, the top three provinces included Jiangsu, Beijing, and Guangdong, accounting for 15.68%, 13.87%, and 9.72% of the total loans, respectively. Moreover, the top five provinces accounted for 52.36% of the national level, while the bottom five provinces only accounting for 0.78% of the national level. The findings confirm that similar to government subsidies, financial institution loans were mainly concentrated in the eastern region of China.

**Table 5:** Distribution of financial institution loans from 2001 to 2018.

Province	Financial institution loans	Percentage (%)	Province	Financial institution loans	Percentage (%)
Jiangsu	7897648	15.68	Hebei	858900	1.71
Beijing	6983639	13.87	Heilongjiang	802131	1.59
Guangdong	4895702	9.72	Guangxi	634010	1.26
Shandong	3350935	6.65	Yunnan	575650	1.14
Zhejiang	3245160	6.44	Shanxi	564479	1.12
Shanghai	3185951	6.33	Jilin	538501	1.07
Anhui	2214723	4.40	Jiangxi	474287	0.94
Sichuan	2186486	4.34	Inner Mongolia	365969	0.73
Tianjin	1620414	3.22	Guizhou	346906	0.69
Fujian	1573527	3.12	Gansu	271417	0.54
Henan	1506167	2.99	Xinjiang	162786	0.32
Hubei	1380774	2.74	Ningxia	97989	0.19
Shaanxi	1347623	2.68	Qinghai	72722	0.14
Liaoning	1087325	2.16	Hainan	61011	0.12
Chongqing	1047445	2.08	Tibet	5543	0.01
Hunan	1002614	1.99			

Unit: ten thousand yuan. Calculated according to the data of each year in China Science and Technology Statistical Yearbook.

## 5. Empirical analysis

Substantive innovation, strategic innovation, and innovation policy concentrated in some regions. To measure the concentration of regional innovation, we applied the Gini coefficient. In this study, the Gini coefficient of substantive innovation, strategic innovation, and innovation policy was close to 1 (S1 Appendix), representing the more geographically concentrated innovation. Thus, it is essential to make a spatial econometric analysis of innovation and innovation policy. Before performing the spatial econometric analysis, it is essential to use the Moran index to obtain the spatial correlation between substantive innovation and strategic innovation. In addition, there exist a strong spatial correlation, and spatial correlation fluctuates with time (S1 Appendix). Hence, the spatial econometric model was set to examine the correlation between innovation and innovation policy.

### 5.1 Innovation policy impact and spatial spillover effect

Following the test method of spatial dependence described elsewhere [65], the model was selected by comparing the significance of the Lagrange multiplier LM-sar and LM-err and the significance of its robust forms robust LM-sar and robust LM-err. The principle is that, if LM-sar is more significant than LM-err, and robust LM-sar is significant while robust LM-err is not, then the SAR model should be selected, else, the SEM model should be selected.

Based on this principle and the Hausman test, we selected the fixed-effect SAR model. In order to eliminate the impact of scale and regional heterogeneity, the innovation, substantive innovation, and strategic innovation is measured by the number of patents, invention patents, utility model and design patents per 1000 R&D full-time equivalent personnel respectively in each region [66,67].

Table 6 shows that the spatial autoregression coefficient of substantive innovation and strategic innovation was 0.545, and 0.636, respectively, which is significant at the 1% level, suggesting the presence of a spatial spillover effect in substantive innovation and strategic innovation of China. Thus, substantive innovation and strategic innovation in one region would affect innovation in another region.

**Table 6:** The estimation results of spatial panel data model.

	Innovation	Substantive innovation	Strategic innovation
lngov	0.199^***^	0.254^***^	0.192^***^
	(0.001)	(0.000)	(0.004)
lnfin	-0.055^***^	-0.101^***^	-0.042^*^
	(0.005)	(0.000)	(0.055)
lngdp	0.714^***^	0.736^***^	0.709^***^
	(0.000)	(0.000)	(0.000)
lnexp	0.062	0.127^***^	0.048
	(0.137)	(0.006)	(0.298)
lnimp	-0.088^**^	-0.040	-0.130^***^
	(0.034)	(0.396)	(0.005)
lnpeo	-0.829^***^	-0.724^***^	-0.823^***^
	(0.000)	(0.000)	(0.000)
eduyear	0.278^**^	0.312^***^	0.291^***^
	(0.000)	(0.000)	(0.000)
*ρ*	0.649^***^	0.545^***^	0.636^***^
	(0.000)	(0.000)	(0.000)
R-squared	0.79	0.84	0.72
Log-L	-83.96	-141.54	-141.38

***, **, * denotes significance at 1%, 5%, 10% level respectively, P values are in parentheses.

Based on the analysis described above, since the coefficients in the spatial econometric model cannot directly reflect the influence of the independent variable on the dependent variable, it is essential to decompose the direct and indirect effects (i.e., spatial spillover effect) of the estimation of innovation policy and other variables, to further measure the spatial spillover effect of the innovation policy and validate its role in the innovation activities.

Table 7 shows that the direct effect and the spatial spillover effect of government subsidies and financial institution loans are significant at the given level of significance, highlighting the significance of focusing on the direct effect and spatial spillover effect of innovation policies at the same time. The direct effect of government subsidies on substantive innovation and strategic innovation was 0.267, 0.199, and the direct effect of financial institution loans on substantive innovation and strategic innovation was –0.104 and –0.045, respectively, suggesting that the direct effect of government subsidies and financial institution loans was higher in substantive innovation than that in strategic innovation. In addition, the spatial spillover effect of government subsidies on substantive innovation and strategic innovation was 0.366 and 0.333, and the spatial spillover effect of financial institution loans on substantive innovation and strategic innovation was –0.122 and –0.078, respectively, suggesting that the spatial spillover effect of government subsidies and financial institution loans was higher in substantive innovation than that in strategic innovation. The innovation policy provides resources, knowledge, and technology for technological innovation of enterprises by supporting R&D input and personnel training to create a good environment [4,5], this helps enterprises overcome the impact of various uncertainties. Thus, enterprises gain sufficient confidence, motivation, and conditions for high-quality substantive innovation. In addition, the innovation policy plays the role of “wind vane” to guide the direction of enterprise investment and decrease the risk of innovation to stimulate the enthusiasm of enterprise to execute substantive innovation and enhance the efficiency and quality of innovation. Moreover, the innovation policy sends a signal to the market that innovation has a good future. Through the role of “wind vane” to attract other social capital to enter the innovation field, the innovation activity could obtain more support, enabling the enterprises to undertake substantive innovation.

**Table 7:** Direct effect and spatial spillover effect.

	Innovation		Substantive innovation		Strategic innovation	
	(1)	(2)	(3)	(4)	(5)	(6)
lngov	0.207^***^	0.361^***^	0.267^***^	0.366^***^	0.199^***^	0.333^**^
	(0.003)	(0.005)	(0.001)	(0.002)	(0.008)	(0.012)
lnfin	-0.058^***^	-0.103^**^	-0.104^***^	-0.122^***^	-0.045^*^	-0.078^*^
	(0.009)	(0.031)	(0.000)	(0.005)	(0.052)	(0.088)
lngdp	0.749^***^	1.325^***^	0.758^***^	0.874^***^	0.738^***^	1.253^***^
	(0.000)	(0.000)	(0.000)	(0.000)	(0.000)	(0.000)
lnexp	0.064	0.115	0.132^**^	0.154^**^	0.052	0.090
	(0.141)	(0.162)	(0.011)	(0.035)	(0.296)	(0.319)
lnimp	-0.094^**^	-0.169^*^	-0.043	-0.052	-0.137^***^	-0.235^*^
	(0.034)	(0.062)	(0.375)	(0.383)	(0.006)	(0.024)
lnpeo	-0.866^***^	-1.539^***^	-0.742^***^	-0.868^***^	-0.858^***^	-1.463^***^
	(0.000)	(0.000)	(0.000)	(0.000)	(0.000)	(0.000)
eduyear	0.290^***^	0.512^***^	0.316^***^	0.367^***^	0.301^***^	0.509^***^
	(0.000)	(0.001)	(0.000)	(0.001)	(0.000)	(0.001)

***, **, * denotes significance at 1%, 5%, 10% level respectively, P values are in parentheses. (1), (3), (5), is direct effect respectively, (2), (4), (6) is spatial spillover effect respectively.

From the impact range, government subsidies are higher than that of financial institution loans, corroborating Guellec and Potterie [68]. From the impact direction, government subsidies exert a significant positive impact on substantive innovation and strategic innovation, whereas financial institution loans exert a significant negative effect on substantive innovation and strategic innovation. The negative impact of financial institution loans on innovation is attributed to different characteristics of government subsidies and financial institution loans, which usually have profitable purposes. Thus, financial institutions are more willing to choose enterprises with short-investment cycle and high-solvency mortgage ability when selecting the loan targets. Typically, the earnings of innovative enterprises are uncertain and have a long research cycle. Meanwhile, the majority of innovative assets are intangible and fail to fulfil the requirements of financial institutions. Besides, innovative enterprises might miss market opportunities because of the strict control of loans approved by financial institutions. Hence, enterprises with strong innovation ability but weak solvency find it challenging to obtain loan support from financial institutions, which decreases the efficiency of using loans from financial institutions [69,70].

Among the control variables, the regional economic development level, exports, and labor quality positively affect innovation, while the imports and scientific and technological personnel input negatively affect innovation. The imports and exports coefficients suggest that foreign competitors cause higher competitive pressure on domestic innovators, whereas the potential foreign markets and the degree of economic opening to the outside world could promote innovation [29]. In addition, the input coefficient of scientific and technological personnel is negative, suggesting that the innovation does not increase proportionally with the number of scientific and technological personnel input, and the efficiency of personnel input is not high. Generally, the regional economic development is accompanied by the flow of production factors and industrial agglomeration, so it can affect the innovation of neighbouring regions through economic factors, technical resources, infrastructure system, and other ways. Hence, most control variables affect the innovation of adjacent areas.

### 5.2 Robustness analysis

#### 5.2.1 Change the policy variables form

Government subsidies not only affect the innovation in the year when the subsidies were provided but also affect innovation over a long period. Thus, the concept of stock was introduced to describe the cumulative impact of policy variables on innovation. The capital stock data of policy variables can be obtained by referring to the calculation methods proposed by Wu [71] and Zhu et al. [12].

$$kgov_{it}=(1-\delta )kgov_{it-1}+gov_{it}$$ (13)

$$kgov_{i,2001}=\frac{gov_{i,2001}}{g_{i}+\delta }$$ (14)

Where *kgov*_*it*_ is the government subsidies stock of region *i* at time *t*,*gov*_*it*_ is the government subsidies of region *i* at time *t*, *kgov*_*i*,2001_ is the government subsidies stock of region *i* at time *t*, *δ* is the depreciation rate, and *g*_*i*_ is the growth rate of government subsidies from 2001 to 2018. According to Zhou et al. [72], the depreciation rate has little influence on the research results. On the basis of summarizing previous studies, the selected depreciation rate in this paper is 15%. The same analysis is used for financial institution loans.

Table 8 shows that the spatial autoregressive coefficient *ρ* of substantive innovation and strategic innovation was 0.479, 0.632, which is significant at the 1% level. Thus, substantive innovation and strategic innovation in one region affect innovation in another region. Table 9 shows that the main variables of the model have still maintained the significance and the same direction of action. The direct effect of government subsidies on substantive innovation and strategic innovation was 0.538 and 0.281, and the direct effect of financial institution loans on substantive innovation and strategic innovation was –0.178 and –0.082, respectively. The findings suggest that the direct effect of government subsidies and financial institution loans is higher in substantive innovation than that in strategic innovation. In addition, the spatial spillover effect of government subsidies on substantive innovation and strategic innovation was 0.483 and 0.455, and the spatial spillover effect of financial institution loans on substantive innovation and strategic innovation was –0.162 and –0.134, respectively. The findings suggest that the spatial spillover effect of government subsidies and financial institution loans is higher in substantive innovation than that in strategic innovation. Moreover, government subsidies exert a significant positive impact on substantive innovation and strategic innovation, whereas financial institution loans exert a significant negative impact on substantive innovation and strategic innovation. In other words, when changing the policy variables form, the same results can be obtained.

**Table 8:** The estimation results of changing the policy variables form.

	Innovation	Substantive innovation	Strategic innovation
lnkgov	0.342^***^	0.532^***^	0.267^***^
	(0.000)	(0.000)	(0.004)
lnkfin	-0.100^***^	-0.174^***^	-0.080^***^
	(0.000)	(0.000)	(0.001)
lngdp	0.694^***^	0.723^***^	0.685^***^
	(0.000)	(0.000)	(0.000)
lnexp	0.061	0.115^***^	0.054
	(0.136)	(0.009)	(0.236)
lnimp	-0.096^**^	-0.049	-0.134^***^
	(0.021)	(0.283)	(0.004)
lnpeo	-0.847^***^	-0.748^***^	-0.833^***^
	(0.000)	(0.000)	(0.000)
eduyear	0.264^***^	0.288^***^	0.278^***^
	(0.000)	(0.000)	(0.000)
*ρ*	0.618^***^	0.479^***^	0.632^***^
	(0.000)	(0.000)	(0.000)
R-squared	0.80	0.86	0.73
Log-L	-77.25	-123.32	-138.92

***, **, * denotes significance at 1%, 5%, 10% level respectively, P values are in parentheses. lnkgov and lnkfin represent the logarithm of government subsidies stock and the logarithm of financial institution loans stock.

**Table 9:** Direct effect and spatial spillover effect of changing the policy variables form.

	Innovation		Substantive innovation		Strategic innovation	
	(1)	(2)	(3)	(4)	(5)	(6)
lnkgov	0.353^***^	0.459^***^	0.538^***^	0.483^***^	0.281^***^	0.455^**^
	(0.000)	(0.001)	(0.000)	(0.000)	(0.005)	(0.011)
lnkfin	-0.103^***^	-0.163^***^	-0.178^***^	-0.162^***^	-0.082^***^	-0.134^**^
	(0.000)	(0.003)	(0.000)	(0.001)	(0.003)	(0.011)
lngdp	0.719^***^	1.126^***^	0.738^***^	0.663^***^	0.714^***^	1.167^***^
	(0.000)	(0.000)	(0.000)	(0.000)	(0.000)	(0.000)
lnexp	0.065	0.102	0.119^**^	0.109^**^	0.056	0.04
	(0.151)	(0.180)	(0.014)	(0.043)	(0.249)	(0.274)
lnimp	-0.100^**^	-0.158^**^	-0.050	-0.047	-0.140^***^	-0.230^**^
	(0.025)	(0.046)	(0.279)	(0.319)	(0.006)	(0.019)
lnpeo	-0.879^***^	-1.386^***^	-0.764^***^	-0.695^***^	-0.871^***^	-1.430^***^
	(0.000)	(0.000)	(0.000)	(0.000)	(0.000)	(0.000)
eduyear	0.275^***^	0.430^***^	0.294^***^	0.265^***^	0.290^***^	0.473^***^
	(0.000)	(0.001)	(0.000)	(0.000)	(0.000)	(0.001)

***, **, * denotes significance at 1%, 5%, 10% level respectively, P values are in parentheses. lnkgov and lnkfin represent the logarithm of government subsidies stock and the logarithm of financial institution loans. (1), (3), (5), is direct effect respectively, (2), (4), (6) is spatial spillover effect respectively.

#### 5.2.2 Lagging of policy variables

Owing to the lagging effect and endogenous of policies, government subsidies and financial institution loans lagging period were introduced into the model, l1gov and l1fin denote the lngov and lnfin, which are with one lag period, respectively.

Table 10 shows that the spatial autoregressive coefficient *ρ* of substantive innovation and strategic innovation was 0.548 and 0.642, respectively, which is significant at the 1% level. Thus, substantive innovation and strategic innovation in one region affect innovation in another region. Table 11 shows that the main variables of the model have still maintained the significance and the same direction of action. In addition, the direct effect of government subsidies and financial institution loans is higher in substantive innovation than that in strategic innovation. Moreover, the spatial spillover effect of government subsidies and financial institution loans is higher in substantive innovation than that in strategic innovation. Besides, government subsidies exert a significant positive impact on substantive innovation and strategic innovation, whereas financial institution loans exert a significant negative impact on substantive innovation and strategic innovation. In other words, when the one lag period of innovation policies is introduced into to model, the same results can be obtained.

**Table 10:** The estimation results of lagging of policy variables.

	Innovation	Substantive innovation	Strategic innovation
l1gov	0.163^***^	0.244^***^	0.133^**^
	(0.007)	(0.000)	(0.045)
l1fin	-0.029^*^	-0.077^***^	-0.013^*^
	(0.098)	(0.000)	(0.081)
lngdp	0.770^***^	0.786^***^	0.758^***^
	(0.000)	(0.000)	(0.000)
lnexp	0.061	0.143^***^	0.039
	(0.150)	(0.002)	(0.409)
lnimp	-0.092^**^	-0.061	-0.127^***^
	(-0.033)	(0.202)	(0.008)
lnpeo	-0.844^***^	-0.749^***^	-0.836^***^
	(0.000)	(0.000)	(0.000)
eduyear	0.277^***^	0.304^***^	0.296^***^
	(0.000)	(0.000)	(0.000)
*ρ*	0.645^***^	0.548^***^	0.642^***^
	(0.000)	(0.000)	(0.000)
R-squared	0.79	0.83	0.72
Log-L	-73.65	-125.47	-129.51

***, **, * denotes significance at 1%, 5%, 10% level respectively, P values are in parentheses. l1gov and l1fin represent the lngov and lnfin which are with one lag period respectively.

**Table 11:** Direct effect and spatial spillover effect of lagging of policy variables.

	Innovation		Substantive innovation		Strategic innovation	
	(1)	(2)	(3)	(4)	(5)	(6)
l1gov	0.174^***^	0.286^**^	0.248^***^	0.290^***^	0.144^**^	0.240^*^
	(0.008)	(0.012)	(0.001)	(0.000)	(0.048)	(0.060)
l1fin	-0.030^*^	-0.054^*^	-0.079^***^	-0.094^**^	-0.014^*^	-0.024^*^
	(0.099)	(0.081)	(0.001)	(0.011)	(0.077)	(0.067)
lngdp	0.809^***^	1.309^***^	0.810^***^	0.949^***^	0.783^***^	1.349^***^
	(0.000)	(0.000)	(0.000)	(0.000)	(0.000)	(0.000)
lnexp	0.062	0.109	0.149^***^	0.177^**^	0.040	0.068
	(0.171)	(0.205)	(0.005)	(0.021)	(0.432)	(0.450)
lnimp	-0.094^**^	-0.165^*^	-0.063	-0.077	-0.134^**^	-0.229^**^
	(0.045)	(0.072)	(0.198)	(0.243)	(0.011)	(0.032)
lnpeo	-0.885^***^	-1.538^***^	-0.773^***^	-0.917^***^	-0.879^***^	-1.503^***^
	(0.000)	(0.000)	(0.000)	(0.000)	(0.000)	(0.000)
eduyear	0.287^***^	0.494^***^	0.313	0.367^***^	0.311^***^	0.527^***^
	(0.000)	(0.000)	(0.000)	(0.000)	(0.000)	(0.001)

***, **, * denotes significance at 1%, 5%, 10% level respectively, P values are in parentheses. (1), (3), (5) is direct effect respectively, (2), (4), (6) is spatial spillover effect respectively. l1gov and l1fin represent the lngov and lnfin which are with one lag period respectively.

#### 5.2.3 Different spatial weight matrices

To avoid the doubt caused by the subjectivity of the spatial weight matrix setting, different spatial weight matrices are applied. Considering the difference in the attenuation speed of the influence intensity, in this part, the spatial weight matrix was established according to the reciprocal third power and the second power of the geographical distance. Considering the economic influence in the space unit, the spatial weight matrix (*WE*) was established per the economic distance [73]. Meanwhile, we also set the human capital spatial weight matrix (*WH*). Through the different spatial weight matrices, we further verified the results obtained above.

The economic distance spatial weight is set as:

WE=W.*E (15)

while

$$E_{ij}=\frac{1}{\left|{\bar{E}}_{i}-{\bar{E}}_{j}\right|}$$ (16)

$${\bar{E}}_{i}=\frac{1}{t_{1}-t_{0}+1}\sum_{t=t_{0}}^{t_{1}}E_{it}$$ (17)

among them *E*_*it*_ is real GDP per capita, representing the level of economic development of region *i* at time *t*.

The human capital spatial weight matrix is set as:

WH=W.*H (18)

while

$$H_{ij}=\frac{1}{\left|{\bar{H}}_{i}-{\bar{H}}_{j}\right|}$$ (19)

$${\bar{H}}_{i}=\frac{1}{t_{1}-t_{0}+1}\sum_{t=t_{0}}^{t_{1}}H_{it}$$ (20)

among them *H*_*it*_ is the R&D full-time equivalent personnel, representing the human capital of region *i* at time *t*.

The results without distinguishing innovation motivation for adopting different spatial weight matrices are shown in Tables 12 and 13. The results with distinguishing innovation motivation for adopting different spatial weight matrices are shown in Tables 14–17. Table 14 shows that when different matrices are adopted, the spatial autoregressive coefficient *ρ* of substantive innovation was 0.256, 0.390, 0.294, and 0.247, respectively. Table 16 shows that when different matrices are adopted, the spatial autoregressive coefficient *ρ* of strategic innovation was 0.384, 0.517, 0.374, and 0.280, respectively. Thus, substantive innovation and strategic innovation in one region affect innovation in another region. Tables 15 and 17 show that the main variables of the model have still maintained the significance and the same direction of action. The direct effect of government subsidies and financial institution loans is higher in substantive innovation than that in strategic innovation. In addition, the spatial spillover effect of government subsidies and financial institution loans is higher in substantive innovation than that in strategic innovation. Moreover, government subsidies exert a significant positive impact on substantive innovation and strategic innovation, whereas financial institution loans exert a significant negative impact on substantive innovation and strategic innovation. In other words, when the different matrices are adopted, the original result will not be changed.

**Table 12:** The estimation results of different spatial weight matrices in innovation.

	(1)	(2)	(3)	(4)
lngov	0.337^***^	0.282^***^	0.302^***^	0.301^***^
	(0.000)	(0.000)	(0.000)	(0.000)
lnfin	-0.049^**^	-0.051^***^	-0.052^***^	-0.049^**^
	(0.013)	(0.009)	(0.008)	(0.019)
lngdp	0.802^***^	0.738^***^	0.816^***^	0.940^***^
	(0.000)	(0.000)	(0.000)	(0.000)
lnexp	0.008	0.027	0.029	0.026
	(0.853)	(0.525)	(0.489)	(0.560)
lnimp	-0.057	-0.063	-0.115^***^	-0.104^**^
	(0.177)	(0.132)	(0.006)	(0.019)
lnpeo	-0.784^***^	-0.799^***^	-0.787^***^	-0.827^***^
	(0.000)	(0.000)	(0.000)	(0.000)
eduyear	0.346^***^	0.310^***^	0.362^***^	0.393^***^
	(0.000)	(0.000)	(0.000)	(0.000)
*ρ*	0.378^***^	0.513^***^	0.405^***^	0.293^***^
	(0.000)	(0.000)	(0.000)	(0.000)
R-squared	0.79	0.79	0.80	0.80
Log-L	-100.56	-91.10	-92.73	-116.68

***, **, * denotes significance at 1%, 5%, 10% level respectively, P values are in parentheses. (1), (2) represent the results of weight matrices which are based on the reciprocal third power and the second power of the geographical distance respectively. (3), (4) represent the results of weight matrices which are based on the economic distance and human capital respectively.

**Table 13:** Direct effect and spatial spillover effect of different spatial weight matrices in innovation.

	(1)	(2)	(3)	(4)	(5)	(6)	(7)	(8)
lngov	0.354^***^	0.188^***^	0.298^***^	0.286^***^	0.316^***^	0.191^***^	0.311^***^	0.120^***^
	(0.000)	(0.000)	(0.000)	(0.000)	(0.000)	(0.000)	(0.000)	(0.001)
lnfin	-0.053^**^	-0.028^**^	-0.054^**^	-0.053^**^	-0.054^**^	-0.033^**^	-0.051^**^	-0.020^**^
	(0.015)	(0.025)	(0.015)	(0.033)	(0.012)	(0.023)	(0.021)	(0.045)
lngdp	0.844^***^	0.449^***^	0.776^***^	0.746^***^	0.858^***^	0.520^**^	0.959^***^	0.371^***^
	(0.000)	(0.000)	(0.000)	(0.000)	(0.000)	(0.000)	(0.000)	(0.000)
lnexp	0.007	0.004	0.031	0.030	0.030	0.018	0.025	0.010
	(0.875)	(0.868)	(0.472)	(0.486)	(0.484)	(0.495)	(0.591)	(0.603)
lnimp	-0.060	-0.032	-0.067	-0.065	-0.120^***^	-0.073^**^	-0.106^**^	-0.041^**^
	(0.184)	(0.201)	(0.131)	(0.152)	(0.009)	(0.020)	(0.024)	(0.049)
lnpeo	-0.823^***^	-0.440^***^	-0.843^***^	-0.814^***^	-0.824^***^	-0.501^***^	-0.848^***^	-0.330^***^
	(0.000)	(0.000)	(0.000)	(0.000)	(0.000)	(0.000)	(0.000)	(0.000)
eduyear	0.362^***^	0.193^***^	0.326^***^	0.314^***^	0.377^***^	0.229^***^	0.402^***^	0.156^***^
	(0.000)	(0.000)	(0.000)	(0.000)	(0.000)	(0.000)	(0.000)	(0.000)

***, **, * denotes significance at 1%, 5%, 10% level respectively, P values are in parentheses. (1), (3), (5), (7) is direct effect respectively, (2), (4), (6), (8) is spatial spillover effect respectively.

**Table 14:** The estimation results of different spatial weight matrices in substantive innovation.

	(1)	(2)	(3)	(4)
lngov	0.401^***^	0.342^***^	0.363^***^	0.363^***^
	(0.000)	(0.000)	(0.000)	(0.000)
lnfin	-0.099^***^	-0.099^***^	-0.092^***^	-0.095^***^
	(0.000)	(0.000)	(0.000)	(0.000)
lngdp	0.933^***^	0.830^***^	0.901^***^	1.005^***^
	(0.000)	(0.000)	(0.000)	(0.000)
lnexp	0.078	0.092^*^	0.103^**^	0.098^**^
	(0.104)	(0.052)	(0.030)	(0.044)
lnimp	-0.023	-0.021	-0.053	-0.047
	(0.634)	(0.660)	(0.264)	(0.331)
lnpeo	-0.690^***^	-0.706^***^	-0.682^***^	-0.709^***^
	(0.000)	(0.000)	(0.000)	(0.000)
eduyear	0.407^***^	0.364^***^	0.409^***^	0.406^***^
	(0.000)	(0.000)	(0.000)	(0.000)
*ρ*	0.256^***^	0.390^***^	0.294^***^	0.247^***^
	(0.000)	(0.000)	(0.000)	(0.000)
R-squared	0.84	0.84	0.85	0.85
Log-L	-161.61	-153.09	-156.09	-164.69

***, **, * denotes significance at 1%, 5%, 10% level respectively, P values are in parentheses. (1), (2) represent the results of weight matrices which are based on the reciprocal third power and the second power of the geographical distance respectively. (3), (4) represent the results of weight matrices which are based on the economic distance and human capital respectively.

**Table 15:** Direct effect and spatial spillover effect of different spatial weight matrices in substantive innovation.

	(1)	(2)	(3)	(4)	(5)	(6)	(7)	(8)
lngov	0.410^***^	0.229^***^	0.352^***^	0.311^***^	0.373^***^	0.245^***^	0.367^***^	0.114^***^
	(0.000)	(0.000)	(0.000)	(0.000)	(0.000)	(0.001)	(0.000)	(0.001)
lnfin	-0.100^***^	-0.032^***^	-0.103^***^	-0.063^***^	-0.095^***^	-0.037^***^	-0.097^***^	-0.031^***^
	(0.000)	(0.005)	(0.000)	(0.002)	(0.000)	(0.007)	(0.000)	(0.007)
lngdp	0.950^***^	0.298^***^	0.854^***^	0.512^***^	0.920^***^	0.358^***^	01.021^***^	0.318^***^
	(0.000)	(0.000)	(0.000)	(0.000)	(0.000)	(0.000)	(0.000)	(0.000)
lnexp	0.081	0.026	0.096^*^	0.058^*^	0.107^**^	0.042^*^	0.100^**^	0.031^*^
	(0.123)	(0.154)	(0.056)	(0.085)	(0.039)	(0.061)	(0.048)	(0.082)
lnimp	-0.020	-0.007	-0.021	-0.014	-0.054	-0.021	-0.048	-0.016
	(0.689)	(0.693)	(0.663)	(0.660)	(0.286)	(0.304)	(0.352)	(0.382)
lnpeo	-0.706^***^	-0.223^***^	-0.726^***^	-0.439^***^	-0.700^***^	-0.274^***^	-0.720^***^	-0.227^***^
	(0.000)	(0.000)	(0.000)	(0.000)	(0.000)	(0.000)	(0.000)	(0.001)
eduyear	0.419^***^	0.132^***^	0.374^***^	0.225^***^	0.419^**^	0.163^***^	0.411^***^	0.129^***^
	(0.000)	(0.000)	(0.000)	(0.000)	(0.000)	(0.000)	(0.000)	(0.000)

***, **, * denotes significance at 1%, 5%, 10% level respectively, P values are in parentheses. (1), (3), (5), (7) is direct effect respectively, (2), (4), (6), (8) is spatial spillover effect respectively.

**Table 16:** The estimation results of different spatial weight matrices in strategic innovation.

	(1)	(2)	(3)	(4)
lngov	0.316^***^	0.266^***^	0.298^***^	0.281^***^
	(0.000)	(0.000)	(0.000)	(0.000)
lnfin	-0.033^*^	-0.036^*^	-0.040^*^	-0.035^*^
	(0.096)	(0.092)	(0.068)	(0.086)
lngdp	0.754^***^	0.708^***^	0.806^***^	0.898^***^
	(0.000)	(0.000)	(0.000)	(0.000)
lnexp	0.002	0.016	0.014	0.014
	(0.961)	(0.727)	(0.760)	(0.776)
lnimp	-0.096^**^	-0.104^**^	-0.156^***^	-0.145^***^
	(0.038)	(0.023)	(0.001)	(0.003)
lnpeo	-0.769^***^	-0.785^***^	-0.794^***^	-0.832^***^
	(0.000)	(0.000)	(0.000)	(0.000)
eduyear	0.339^***^	0.308^***^	0.369^***^	0.402^***^
	(0.000)	(0.000)	(0.000)	(0.000)
*ρ*	0.384^***^	0.517^***^	0.374^***^	0.280^***^
	(0.000)	(0.000)	(0.000)	(0.000)
R-squared	0.72	0.72	0.73	0.72
Log-L	-149.10	-143.06	-151.96	-168.45

***, **, * denotes significance at 1%, 5%, 10% level respectively, P values are in parentheses. (1), (2) represent the results of weight matrices which are based on the reciprocal third power and the second power of the geographical distance respectively. (3), (4) represent the results of weight matrices which are based on the economic distance and human capital respectively.

**Table 17:** Direct effect and spatial spillover effect of different spatial weight matrices in strategic innovation.

	(1)	(2)	(3)	(4)	(5)	(6)	(7)	(8)
lngov	0.333^**^	0.183^***^	0.281^***^	0.268^***^	0.305^***^	0.165^***^	0.287^***^	0.104^***^
	(0.000)	(0.000)	(0.000)	(0.001)	(0.000)	(0.001)	(0.000)	(0.003)
lnfin	-0.035^*^	-0.019^*^	-0.038^*^	-0.037^*^	-0.043^*^	-0.023^*^	-0.036^*^	-0.013^*^
	(0.075)	(0.087)	(0.094)	(0.098)	(0.068)	(0.097)	(0.062)	(0.060)
lngdp	0.789^***^	0.434^***^	0.749^***^	0.717^***^	0.835^***^	0.454^**^	0.920^***^	0.334^***^
	(0.000)	(0.000)	(0.000)	(0.000)	(0.000)	(0.000)	(0.000)	(0.000)
lnexp	0.002	0.001	0.018	0.018	0.015	0.009	0.012	0.004
	(0.975)	(0.982)	(0.720)	(0.711)	(0.756)	(0.755)	(0.816)	(0.824)
lnimp	-0.101^**^	-0.056^*^	-0.110^**^	-0.107^**^	-0.160^***^	-0.087^***^	-0.146^***^	-0.053^**^
	(0.049)	(0.067)	(0.028)	(0.045)	(0.003)	(0.009)	(0.007)	(0.025)
lnpeo	-0.810^***^	-0.446^***^	-0.830^***^	-0.799^***^	-0.821^***^	-0.448^***^	-0.849^***^	-0.311^***^
	(0.000)	(0.000)	(0.000)	(0.000)	(0.000)	(0.000)	(0.000)	(0.000)
eduyear	0.359^***^	0.198^***^	0.325^***^	0.312^***^	0.383^***^	0.208^***^	0.409^***^	0.149^***^
	(0.000)	(0.000)	(0.000)	(0.000)	(0.000)	(0.000)	(0.000)	(0.001)

***, **, * denotes significance at 1%, 5%, 10% level respectively, P values are in parentheses. (1), (3), (5), (7) is direct effect respectively, (2), (4), (6), (8) is spatial spillover effect respectively.

#### 5.2.4 Extended model

Innovation is a continuous process, and the innovation accumulation and performance of the previous period could affect the performance of the current period. Besides, although some control variables that affect regional innovation have been set, some crucial variables might be omitted, which could affect the stability of the estimation results. To further reveal the dynamic dependence between the regional innovation and the innovation policy, we joined ln *pwp*_*it*−1_, ln *pfm*_*it*−1_, and ln *psw*_*it*−1_ in the model. In addition, a dynamic spatial panel model was established for the data provided above.

$$\begin{array}{l} \text{ln}\;pwp_{it}=\alpha +\tau \;\text{ln}\;pwp_{it-1}+\varphi \sum_{j=1}^{n}w_{ij}\;\text{ln}\;pwp_{jt-1}+\rho \sum_{j=1}^{n}w_{ij}\;\text{ln}\;pwp_{jt}\\ +\beta_{1}\;\text{ln}\;gov_{it}+\beta_{2}\;\text{ln}\;fin_{it}+\sum_{k}\delta_{k}x_{kit}+\gamma_{1}\sum_{j=1}^{n}w_{ij}\;\text{ln}\;gov_{it}+\gamma_{2}\sum_{j=1}^{n}w_{ij}\;\text{ln}\;fin_{it}\\ +\sum_{k}\theta_{k}\sum_{j=1}^{n}w_{ij}x_{kit}+\mu_{it} \end{array}$$ (21)

$$\begin{array}{l} \text{ln}\;pfm_{it}=\alpha +\tau \;\text{ln}\;pfm_{it-1}+\varphi \sum_{j=1}^{n}w_{ij}\;\text{ln}\;fm_{jt-1}+\rho \sum_{j=1}^{n}w_{ij}\;\text{ln}\;fm_{jt}\\ +\beta_{1}\;\text{ln}\;gov_{it}+\beta_{2}\;\text{ln}\;fin_{it}+\sum_{k}\delta_{k}x_{kit}+\gamma_{1}\sum_{j=1}^{n}w_{ij}\;\text{ln}\;gov_{it}+\gamma_{2}\sum_{j=1}^{n}w_{ij}\;\text{ln}\;fin_{it}\\ +\sum_{k}\theta_{k}\sum_{j=1}^{n}w_{ij}x_{kit}+\mu_{it} \end{array}$$ (22)

$$\begin{array}{l} \text{ln}\;psw_{it}=\alpha +\tau \;\text{ln}\;psw_{it-1}+\varphi \sum_{j=1}^{n}w_{ij}\;\text{ln}\;psw_{jt-1}+\rho \sum_{j=1}^{n}w_{ij}\;\text{ln}\;psw_{jt}\\ +\beta_{1}\;\text{ln}\;gov_{it}+\beta_{2}\;\text{ln}\;fin_{it}+\sum_{k}\delta_{k}x_{kit}+\gamma_{1}\sum_{j=1}^{n}w_{ij}\;\text{ln}\;gov_{it}+\gamma_{2}\sum_{j=1}^{n}w_{ij}\;\text{ln}\;fin_{it}\\ +\sum_{k}\theta_{k}\sum_{j=1}^{n}w_{ij}x_{kit}+\mu_{it} \end{array}$$ (23)

where, ln *pwp*_*it*−1_, ln *pfm*_*it*−1_, ln *psw*_*it*−1_ represent the explained variable with a lag of one period.

Table 18 shows that substantive innovation and strategic innovation of the last period exert a significant positive impact on the current period, suggesting a cumulative effect of substantive innovation and strategic innovation. The spatial autoregressive coefficient of substantive innovation and strategic innovation was 0.400 and 0.405, respectively, which is significant at the 1% level. Thus, substantive innovation and strategic innovation in one region affect innovation in another region. Table 19 shows that the main variables of the model have still maintained the significance and the same direction of action. The direct effect of government subsidies and financial institution loans is higher in substantive innovation than that in strategic innovation. In addition, the spatial spillover effect of government subsidies and financial institution loans is higher in substantive innovation than that in strategic innovation. Government subsidies exert a significant positive impact on substantive innovation and strategic innovation, whereas financial institution loans exert a significant negative impact on substantive innovation and strategic innovation. In other words, when considering the dynamic dependence between regional innovation and innovation policy, the same results can be obtained.

**Table 18:** The estimation results of dynamic spatial panel data model.

	Innovation	Substantive innovation	Strategic innovation
L1. lnpwp	0.723^***^		
	(0.000)		
L1. lnpfm		0.724^***^	
		(0.000)	
L1. lnpsw			0.742^***^
			(0.000)
WL1.lnpwp	-0.191^***^		
	(0.048)		
WL1.lnpfm		-0.220^**^	
		(0.019)	
WL1.lnpsw			-0.094
			(0.375)
lngov	0.112^***^	0.154^**^	0.110^**^
	(0.009)	(0.022)	(0.015)
lnfin	-0.042^***^	-0.057^***^	-0.035^**^
	(0.002)	(0.000)	(0.019)
lngdp	0.296^***^	0.282^***^	0.268^***^
	(0.000)	(0.000)	(0.000)
lnexp	-0.025	0.034	-0.046
	(0.372)	(0.290)	(0.154)
lnimp	0.0007	-0.033	0.010
	(0.980)	(0.306)	(0.761)
lnpeo	-0.382^***^	-0.325^***^	-0.392
	(0.000)	(0.000)	(0.000)
eduyear	0.063^*^	0.117^***^	0.037
	(0.096)	(0.006)	(0.386)
*ρ*	0.437^***^	0.400^***^	0.405^***^
	(0.000)	(0.000)	(0.000)
R-squared	0.82	0.93	0.88
Log-L	139.40	75.34	76.56

***, **, * denotes significance at 1%, 5%, 10% level respectively, P values are in parentheses. L1. lnpwp denotes the explained variable ln *pwp*_*it*_ with a lag of one period, WL1.lnpwp denotes the cross product of ln *pwp*_*it*−1_ and spatial weight matrix. L1. lnpfm denotes the explained variable ln *pfm*_*it*_ with a lag of one period, WL1.lnpfm denotes the cross product of ln *pfm*_*it*−1_ and spatial weight matrix. L1. lnpsw denotes the explained variable ln *psw*_*it*_ with a lag of one period, WL1.lnpsw denotes the cross product of ln *psw*_*it*−1_ and spatial weight matrix.

**Table 19:** Direct effect and spatial spillover effect in dynamic spatial panel data model.

	Innovation		Substantive innovation		Strategic innovation	
	(1)	(2)	(3)	(4)	(5)	(6)
lngov	0.119^**^	0.093^**^	0.160^**^	0.114^*^	0.124^***^	0.085^*^
	(0.004)	(0.041)	(0.029)	(0.069)	(0.008)	(0.054)
lnfin	-0.043^***^	-0.034^**^	-0.058^***^	-0.039^**^	-0.036^**^	-0.025^*^
	(0.001)	(0.033)	(0.000)	(0.025)	(0.014)	(0.077)
lngdp	0.301^***^	0.235^**^	0.287^***^	0.192^**^	0.272	0.186^**^
	(0.000)	(0.007)	(0.000)	(0.015)	(0.000)	(0.016)
lnexp	-0.026	-0.020	0.036	0.024	-0.046	-0.032
	(0.373)	(0.424)	(0.271)	(0.335)	(0.152)	(0.227)
lnimp	0.001	0.002	-0.034	-0.023	0.009	-0.006
	(0.960)	(0.951)	(0.306)	(0.361)	(0.785)	(0.810)
lnpeo	-0.390^***^	-0.306^***^	-0.330^***^	-0.223^***^	-0.399^***^	-0.275^***^
	(0.000)	(0.005)	(0.000)	(0.009)	(0.000)	(0.008)
eduyear	0.067^*^	0.052	0.123^***^	0.082^***^	0.041	0.027
	(0.075)	(0.138)	(0.004)	(0.042)	(0.334)	(0.404)

***, **, * denotes significance at 1%, 5%, 10% level respectively, P values are in parentheses. The effect decomposition of dynamic spatial panel has short-term effect and long-term effect, while short-term effect and static spatial panel effect have the same form, so only short-term effect is disclosed here. (1), (3), (5) is direct effect respectively, (2), (4), (6) is spatial spillover effect respectively.

## 6. Conclusions

By the spatial econometric model, we empirically investigated the direct effect and spillover effect of the innovation policy in the distribution and dynamic evolution of the regional innovation in China from the perspective of innovation motivation. Meanwhile, different innovation policy tools should be distinguished to determine which innovation policy is more effective. Moreover, the stock form of policy variables, lagging policy variables, different spatial weight matrices, and dynamic spatial panel model were applied to test the robustness of results.

Different from the previous classification of innovation from innovation content or innovation intensity, this paper classifies innovation behavior from the perspective of innovation motivation. At the same time, from the perspective of innovation motivation, the direct effect and spatial spillover effect of innovation policy on innovation are integrated into a unified analysis framework. First, this study reports geographic concentration and spatial correlation in substantive innovation and strategic innovation. Substantive innovation and strategic innovation in one region could affect innovation in another region. As the innovation policy provides resources, knowledge, and technology to help enterprises overcome the impact of various uncertainties. Innovators can have sufficient confidence, motivation, and conditions for high-quality substantive innovation. In addition, innovation policy plays the role of “wind vane”, which can guide enterprises to invest and reduce the risk of innovation failure. Moreover, the innovation policy can also send a signal to the market that innovation has a good future, which can attract other social capital to enter the innovation field. Thus, enterprises can undertake substantive innovation. Therefore, when put the direct and spillover effect of innovation into unified framework, the direct effect and spatial spillover effect of the innovation policy are higher in substantive innovation than that in strategic innovation. As the financial institutions are more willing to choose enterprises with short-investment cycle and high-solvency mortgage ability when selecting the loan targets in regard of the profitable purposes, financial institution loans exert a significant negative impact on substantive innovation and strategic innovation, whereas government subsidies exert a significant positive impact on substantive innovation and strategic innovation.

Compared to the previous studies, the contributions of this study are to classify innovation behavior from the perspective of innovation motivation and focused on the impact of policy from the perspective of innovation motivation, especially put the direct effect and spatial spillover effect of innovation policy into a unified analysis framework from the perspective of innovation motivation. Thus, the government will refine and screen the difficulty, depth and potential value of innovation activities and encourage more enterprises to obtain core competitiveness through substantive innovation. Meanwhile, the government will establish a new standard of innovation policy support, offer more support to high-quality substantive innovation, and then give full play to the efficacy of the innovation policy. In addition, different innovation policy tools are considering to analyse the impact of innovation policy. Through considering different policy tools, the government can identify which innovation policy is more effective and this will promote the sustainable development of innovation.

Based on this model and the reported empirical findings, further studies should focus on the innovation of cities, counties, universities, and scientific research institution, to enrich research in related fields. Notably, the major limitation of this study was that substantive innovation was measured by the invention patent and strategic innovation was measured by utility model and design patent; using them to measure innovation motivation could be rough. We hope this limitation would be addressed in future studies.

## Supporting information

S1 Dataset The data were obtained from the http://data.cnki.net/. (XLSX)

S1 Appendix (DOCX)
