# Supplementary material for: Regional innovation distribution and its dynamic evolution: Policy impact and spillover effect—Based on the perspective of innovation motivation
Source: PLoS One. 2020 Jul 10;15(7):e0235828. doi: 10.1371/journal.pone.0235828 (PMC7351176; doi:10.1371/journal.pone.0235828)
Supplement: S1 Appendix — (DOCX) [file pone.0235828.s002.docx]

# Appendix

1. Gini coefficient

The Gini coefficient was calculated as follows:

$G=\frac{1}{2N^{2}\bar{y}\sum_{i=1}^{N} \sum_{j=1}^{N} |y_{i}-y_{j}|}$ (24)

where *N* denotes the number of regions and $\bar{y}$ implies the average value of innovation. Zhang and Li [74] suggested that the closer the Gini coefficient is close to 1, the more geographically concentrated the innovation is. The same step was applied to evaluate the Gini coefficient of government subsidies and financial institution loans.

**Table I Gini index**

|  | **Innovation** | **Substantive**  **innovation** | **Strategic**  **innovation** | **Government Subsidies** | **Financial institution loans** |
| --- | --- | --- | --- | --- | --- |
| 2001 | 0.547 | 0.547 | 0.559 | 0.576 | 0.509 |
| 2002 | 0.562 | 0.538 | 0.579 | 0.580 | 0.574 |
| 2003 | 0.570 | 0.562 | 0.587 | 0.573 | 0.571 |
| 2004 | 0.585 | 0.579 | 0.601 | 0.583 | 0.577 |
| 2005 | 0.614 | 0.599 | 0.629 | 0.597 | 0.596 |
| 2006 | 0.626 | 0.618 | 0.638 | 0.589 | 0.584 |
| 2007 | 0.642 | 0.628 | 0.655 | 0.589 | 0.599 |
| 2008 | 0.643 | 0.622 | 0.662 | 0.581 | 0.568 |
| 2009 | 0.646 | 0.609 | 0.665 | 0.593 | 0.572 |
| 2010 | 0.641 | 0.615 | 0.658 | 0.616 | 0.562 |
| 2011 | 0.637 | 0.617 | 0.653 | 0.612 | 0.601 |
| 2012 | 0.640 | 0.607 | 0.662 | 0.607 | 0.597 |
| 2013 | 0.623 | 0.600 | 0.645 | 0.605 | 0.584 |
| 2014 | 0.594 | 0.582 | 0.611 | 0.611 | 0.640 |
| 2015 | 0.578 | 0.580 | 0.589 | 0.611 | 0.613 |
| 2016 | 0.583 | 0.583 | 0.598 | 0.609 | 0.613 |
| 2017 | 0.580 | 0.585 | 0.590 | 0.596 | 0.596 |
| 2018 | 0.587 | 0.594 | 0.593 | 0.597 | 0.576 |

As shown in Table I, high geographic concentration was observed in regional substantive innovation, strategic innovation, and innovation policy distribution. During 2001–2018, the Gini coefficient of substantive innovation increased from 0.547 to 0.587, that of strategic innovation increased from 0.547 to 0.594, that of substantive innovation increased from 0.559 to 0.593, that of government subsidies increased from 0.576 to 0.597, and that of loans from financial institutions increased from 0.509 to 0.576. Hence, based on the Gini coefficient, high geographic concentration was noted in the distribution of substantive innovation, strategic innovation, and innovation policy.

2. Moran index

The Moran index was calculated as follows:

$Moran'sI=\frac{\sum_{i=1}^{n} \sum_{j=1}^{n} w_{ij}(y_{i}-\bar{y})(y_{j}-\bar{y})}{S^{2}\sum_{i=1}^{n} \sum_{j=1}^{n} w_{ij}}$ (25)

where,$w_{ij}$ is the $(i,j)$element of spatial weight matrix$W$, $y_{i}$ is the observed value of the $i$th region, $\bar{y}=\frac{1}{n}\sum_{i=1}^{n} y_{i}$, and $S^{2}=\frac{1}{n}\sum_{i=1}^{n} (y_{i}-\bar{y})^{2}$, The value range of$I$is $\left[-1,1\right]$,when $I>0$represents positive spatial correlation, and$I<0$ represents negative spatial correlation. The results are displayed in Table II.

**Table II Moran index of different type of innovation**

|  | **Innovation** | **Substantive**  **innovation** | **Strategic**  **innovation** |
| --- | --- | --- | --- |
| 2001 | 0.018  (0.053) | 0.026  (0.044) | 0.012  (0.059) |
| 2002 | 0.025  (0.056) | 0.037  (0.023) | 0.023  (0.053) |
| 2003 | 0.020  (0.079) | 0.041  (0.020) | 0.016  (0.091) |
| 2004 | 0.012  (0.061) | 0.041  (0.022) | 0.016  (0.095) |
| 2005 | 0.025  (0.054) | 0.031  (0.047) | 0.021  (0.068) |
| 2006 | 0.022  (0.070) | 0.031  (0.088) | 0.023  (0.067) |
| 2007 | 0.035  (0.029) | 0.038  (0.090) | 0.040  (0.021) |
| 2008 | 0.047  (0.011) | 0.033  (0.078) | 0.051  (0.007) |
| 2009 | 0.047  (0.009) | 0.021  (0.094) | 0.048  (0.007) |
| 2010 | 0.059  (0.002) | 0.018  (0.095) | 0.066  (0.001) |
| 2011 | 0.047  (0.005) | 0.018  (0.085) | 0.050  (0.003) |
| 2012 | 0.050  (0.002) | 0.029  (0.033) | 0.051  (0.002) |
| 2013 | 0.051  (0.003) | 0.039  (0.014) | 0.050  (0.004) |
| 2014 | 0.051  (0.005) | 0.050  (0.006) | 0.044  (0.011) |
| 2015 | 0.056  (0.005) | 0.057  (0.005) | 0.045  (0.012) |
| 2016 | 0.058 | 0.065 | 0.046 |
|  | (0.004) | (0.002) | (0.011) |
| 2017 | 0.040 | 0.051 | 0.029 |
|  | (0.017) | (0.008) | (0.038) |
| 2018 | 0.039 | 0.063 | 0.025 |
|  | (0.016) | (0.003) | (0.046) |

Note: P values are in parentheses.

Table II shows that substantive innovation and strategic innovation has a strong spatial correlation, which fluctuates with time. Hence, the spatial econometric model could be used to analyse the correlation between innovation and innovation policy.

Rferences

74. Zhang YM, Li K. Research on the spatial dependence of Chinese province--level innovation output. Studies in Science of Science, 2008, (3): 215-221.
